# Possible Reactivation of SARS-CoV-2 in a Patient with Acute Myeloid Leukemia Undergoing Allogeneic Hematopoietic Stem Cell Transplantation: a Case Report

**DOI:** 10.1007/s42399-021-01020-0

**Published:** 2021-07-20

**Authors:** Vera Dalla Via, Matthias von Rotz, Veronika Bättig, Karoline Leuzinger, Hans H. Hirsch, Jakob Passweg, Georg Stüssi, Michael Medinger

**Affiliations:** 1 Division of Hematology, Department of Medicine, University Hospital Basel and University of Basel, Petersgraben 4, CH-4031 Basel, Switzerland; 2 Division of Infectious Diseases and Hospital Epidemiology, University Hospital Basel and University of Basel, Basel, Switzerland; 3 Clinical Virology, Laboratory Medicine, University Hospital Basel, Basel, Switzerland; 4 Transplantation and Clinical Virology, Department of Biomedicine, University of Basel, Basel, Switzerland; 5 Oncological Institute of Italian Switzerland, Bellinzona, Switzerland; 6 Division of Internal Medicine, Department of Medicine, University Hospital Basel and University of Basel, Basel, Switzerland

**Keywords:** Acute myeloid leukemia, Allogeneic hematopoietic stem cell transplantation, COVID-19, SARS-CoV-2

## Abstract

Reactivation or reinfection cases of SARS-CoV-2 are known but there is scarce evidence about reactivation in immunocompromised patients. Here, we report the case of a 61-year-old male undergoing a conditioning regimen with fludarabine, cyclophosphamide, and 2-Gy total body irradiation in preparation of a haplo-identical allogeneic hematopoietic stem cell transplantation (allo-HSCT) for acute myeloid leukemia (AML). He received the first dose of a COVID-19 vaccine 6 weeks prior allo-HSCT and was hospitalized a month prior because of a COVID-19 bilateral pneumonia. On discharge, he showed two negative SARS-CoV-2 nasopharyngeal PCR swabs as well as a high SARS-CoV-2 antibody titer. On admission for allo-HSCT, he tested negative for SARS-CoV-2 again. Conditioning with fludarabine, cyclophosphamide, and 2-Gy total body irradiation was started and the patient developed lymphopenia. During his hospital stay, he tested positive for SARS-CoV-2 in a PCR test twice but remained asymptomatic. The conditioning regimen was continued as planned. Later during his stay, the patient showed undetectable SARS-CoV-2 load four times. This case documents possible reactivation of SARS-CoV-2 and raises questions about reactivation risks among recipients of stem cell transplants and other immunocompromised patients.

## Introduction

Until now, SARS-CoV-2 has infected about 170 million people worldwide with almost 3.5 million deaths according to the last WHO epidemiological report (https://www.who.int). The evidence about reactivation of SARS-CoV-2 after infection is scarce. Coppola et al. [1] reported a patient who showed a re-positive SARS-CoV-2 swab after cured COVID-19 pneumonia and two negative swabs. Ye et al. reported 5 cases of oligosymptomatic SARS-CoV-2 reactivation [2]. There are even more sporadic data on SARS-CoV-2 reactivation or prolonged viral shedding in immunosuppressed patients. Lancman et al. report one patient with a severe SARS-CoV-2 reactivation after receiving chemo-immunotherapy for a B-cell acute lymphoblastic leukemia [3].

## Case Presentation

Here, we report a case of a probable reactivation of SARS-CoV-2 in an immunosuppressed patient who previously had COVID-19 and who had received one dose of COVID-19 mRNA vaccination (BNT162b2, BioNTech/Pfizer). A 61-year-old male suffering from acute myeloid leukemia (AML) was admitted to our center for a planned allogeneic hematopoietic stem cell transplantation (allo-HSCT). AML was diagnosed in March 2020. The patient received no intensive induction chemotherapy because of coronary heart disease as comorbidity. The patient received 11 cycles of the hypomethylating agent decitabine combined with the BCL2-inhibitor venetoclax until February 2021. The AML was morphological in complete remission but with molecular persistence of NPM1. Thus, allo-HSCT was planned. In February 2021, the patient had received the first dose of a COVID-19 mRNA vaccine (BNT162b2, BioNTech/Pfizer). Two weeks later, when the beginning of the 12^th^ cycle of chemotherapy was planned, he had presented to another center with myalgia, fever, and gastro-intestinal symptoms, without respiratory symptoms. A naso-oropharyngeal swab was tested SARS-CoV-2 positive by PCR (Table 1). During his stay, the patient did not develop any significant respiratory symptoms and did not require oxygen supplementation. CT scan showed subpleural ground glass infiltration compatible with COVID-19 pneumonia with a very limited extension. He was treated with symptomatic therapy and standard antithrombotic prophylaxis. The clinical course was favorable and the patient was discharged after 11 days. Two following naso-oropharyngeal swabs, performed 7 and 10 days after diagnosis, respectively, were negative. At the same time, anti-SARS-CoV-2 IgG with a high titer of 743 AU/ml was detected; IgM was equivocal. Since the patient had developed the infection 2 weeks after receiving the first dose of the vaccine and since SARS-CoV-2 IgG antibodies were already positive, we decided not to administer the second dose of the COVID-19 vaccine, assuming that the patient would acquire natural immunity after the infection. Due to the good general condition of the patient and the molecular leukemia persistence, we decided to proceed to allo-HSCT without any delay. On admission at our center, the patient was asymptomatic and physical examination was normal. Laboratory tests showed mild anemia and thrombocytopenia with normal CRP and without signs of a coagulopathy. The AML was in morphological complete remission prior to allo-HSCT. A CT showed no residual signs for pulmonary infection. SARS-CoV-2 was undetectable in the naso-oropharyngeal swab. We started the planned conditioning regimen with fludarabine, cyclophosphamide, and 2-Gy total body irradiation (TBI) which was well-tolerated. The GvHD prophylaxis consisted of high-dose cyclophosphamide post-transplant, tacrolimus, and mycophenolate mofetil. The stem cell source was bone marrow from his haplo-identical son. A few days after starting conditioning, the patient developed lymphopenia (Table 1) in addition to the already known anemia and thrombocytopenia. A new, routinely performed SARS-CoV-2 saliva PCR on day −4 before HSCT came out to be positive, with a semi-quantitative CT value of 35.9 (cycle threshold). The antibody testing was repeated, showing 136 U/ml anti-SARS-CoV-2 spike antibodies (normal range < 0.7 U/ml) and 31.9 U/ml anti-SARS-CoV-2 nucleocapsid-antibodies (normal range ≤ 1 COI). The antibody testing was performed with the electro-chemiluminescence immunoassay (ECLIA) method. The patient was afebrile and asymptomatic and the inflammation markers were normal. We continued the conditioning chemotherapy. Since a worsening of the immune situation was expected, we administered one unit of convalescent plasma. A confirmatory naso-pharyngeal swab PCR tested SARS-CoV-2 positive. The quantitative viral load was however below the limit of detection (< 1000 copies/ml). Two days later, a monitoring SARS-CoV-2 quantitative PCR test showed again a viral load of <1000 copies/ml and the isolation was ended according to the internal guidelines. In addition, the following SARS-CoV-2 quantitative PCR tests showed viral loads of < 1000 copies/ml (Table 1). The neutrophil engraftment (> 0.5 G/l) occurred on day +23 and the patient discharged on day +31. Lymphopenia persisted until discharge (0.02 G/l). Two months after allo-HSCT, the patient is still in complete molecular remission (NPM1A negative in peripheral blood and bone marrow) without signs of acute graft-versus-host disease.

**Table 1:** Course of SARS-CoV-2 infection and possible SARS-CoV-2 reactivation of the patient with acute myeloid leukemia

Date All in 2021	26.2.	07.3.	09.3.	15.3.	26.3.	29.3.	07.4.	10.4.	12.4.	13.4.	15.4.	16.4.	17.4.	20.4.	30.04.	10.05.
SARS-CoV-2 vaccination (BNT162b2, BioNTech/Pfizer)	X															
Begin symptoms		Myalgia, diarrhea, fatigue, nausea														
COVID pneumonia			X													
SARS-CoV-2 PCR nasopharyngeal			Pos.	Pos.	Neg.	Neg.	Neg.			Pos.						
SARS-CoV-2 PCR saliva									Pos.				Neg.	Neg.	Neg.	Neg.
SARS-CoV-2 quantitative PCR nasopharyngeal										< 1000 copies/ml	< 1000 copies/ml			< 1000 copies/ml	< 1000 copies/ml	< 1000 copies/ml
SARS-CoV-2 IgG				IgG : 743 AU/ml (normal range < 50 AU/ml)						Anti-spike: 136 U/ml (normal range < 0.7 U/ml), anti-nucleocapsid 31.9 COI (normal range ≤ 1)						
Begin conditioning fludarabine, cyclophosphamide, and 2-Gy total body irradiation								X								
Date allo-HSCT												X				
Absolute lymphocyte count (G/l)							1.14	1.27	0.23	0.17	0.07	0.03	0.05	0.02	0.02	0.02
Neutrophil engraftment (> 0.5 G/l)																X

## Discussion

Re-positive SARS-CoV-2 tests in recovered COVID-19 patients are known [4, 5]. The causes of reactivation remain unclear and might include reinfection, prolonged or intermittent viral shedding, and laboratory errors. Patients undergoing hematopoietic stem cell transplantation are at increased risk for infections with community-acquired respiratory viruses (CARVs) [5]. Coronaviruses are among the most frequently detected CARVs in these patients [6, 7]. Prolonged shedding for more than 21 days is common in these patients and is related with immunodeficiency of the host and treatment-associated factors, including conditioning regimens [8]. Lu et al. [9] observed 14% of re-positive SARS-CoV-2 tests in a population of 619 patients. In this population, it was not possible to isolate the virus on samples from re-positive patients and multiple PCR sequencing showed highly degraded genomes, suggesting that the re-positive tests might be caused by intermittent shedding of viral RNA fragments from the first infection. The viral shedding in sputum lasts longer than in the upper respiratory tract tests [10]. Patients who show negative SARS-CoV-2 throat swabs might therefore still be shedding in the lower respiratory tract [11].

Our patient had been isolated under strict precaution measures since his admission to our center; therefore, a reinfection is unlikely. Since the PCR for SARS-CoV-2 was positive on two separate occasions, a false positive result is also unlikely. We interpreted the re-positive tests most likely as an asymptomatic flare-up, possibly caused by a prolonged or intermittent shedding and favored by immunosuppression because of the AML and chemotherapy conditioning. Contrary to the patient reported by Lancman et al. [3], our patient had not lost his antibodies at the time of “reactivation”, despite his hematologic disorder and lymphopenia. Interestingly, contrary to the severe reactivation reported by Lancman et al. [3], our patient remained asymptomatic during the reactivation. It is however unclear whether there might be a correlation between these two observations. It is also still unclear whether the patient will lose his anti-SARS-CoV-2 antibodies after the allogeneic stem cell transplantation.

In a large study of 382 COVID-19 patients after HSCT by Ljungman et al. [12], the following risk factors for a worse outcome of COVID-19 were found: older age, intensive care unit need, and a moderate/high immunodeficiency index, whereas underlying diagnosis, time from HSCT, GvHD, or ongoing immunosuppression did not significantly impact overall survival.

## Conclusions

This case documents the possible reactivation of SARS-CoV-2 in patients affected by hematologic malignancies and receiving immunosuppressive medications and raises questions about potential reactivation risks and efficacy of immune response against SARS-CoV-2 in stem cell transplantation recipients, as well as about precaution, isolation measures, and optimal timing of HSCT after COVID-19 infection. Patients with a history of COVID-19 infection and planned allo-HSCT should be regularly monitored for SARS-COV-2 reactivation by PCR tests.
